# Plasma Microbial Cell-Free DNA Sequencing from over 15,000 Patients Identified a Broad Spectrum of Pathogens

**DOI:** 10.1128/jcm.01855-22

**Published:** 2023-07-13

**Authors:** Sarah Y. Park, Eliza J Chang, Nathan Ledeboer, Kevin Messacar, Martin S. Lindner, Shivkumar Venkatasubrahmanyam, Judith C. Wilber, Marla Lay Vaughn, Sivan Bercovici, Bradley A. Perkins, Frederick S. Nolte

**Affiliations:** a Karius, Redwood City, California, USA; b Medical College of Wisconsin, Milwaukee, Wisconsin, USA; c University of Colorado, Children’s Hospital Colorado, Aurora, Colorado, USA; National Institute of Allergy and Infectious Diseases

**Keywords:** microbial cell-free DNA, high-throughput nucleic acid sequencing, liquid biopsy for infectious diseases, metagenomics

## Abstract

Microbial cell-free DNA (mcfDNA) sequencing is an emerging infectious disease diagnostic tool which enables unbiased pathogen detection and quantification from plasma. The Karius Test, a commercial mcfDNA sequencing assay developed by and available since 2017 from Karius, Inc. (Redwood City, CA), detects and quantifies mcfDNA as molecules/μL in plasma. The commercial sample data and results for all tests conducted from April 2018 through mid-September 2021 were evaluated for laboratory quality metrics, reported pathogens, and data from test requisition forms. A total of 18,690 reports were generated from 15,165 patients in a hospital setting among 39 states and the District of Columbia. The median time from sample receipt to reported result was 26 h (interquartile range [IQR] 25 to 28), and 96% of samples had valid test results. Almost two-thirds (65%) of patients were adults, and 29% at the time of diagnostic testing had ICD-10 codes representing a diverse array of clinical scenarios. There were 10,752 (58%) reports that yielded at least one taxon for a total of 22,792 detections spanning 701 unique microbial taxa. The 50 most common taxa detected included 36 bacteria, 9 viruses, and 5 fungi. Opportunistic fungi (374 Aspergillus spp., 258 Pneumocystis jirovecii, 196 *Mucorales*, and 33 dematiaceous fungi) comprised 861 (4%) of all detections. Additional diagnostically challenging pathogens (247 zoonotic and vector-borne pathogens, 144 Mycobacterium spp., 80 *Legionella* spp., 78 systemic dimorphic fungi, 69 *Nocardia* spp., and 57 protozoan parasites) comprised 675 (3%) of all detections. This is the largest reported cohort of patients tested using plasma mcfDNA sequencing and represents the first report of a clinical grade metagenomic test performed at scale. Data reveal new insights into the breadth and complexity of potential pathogens identified.

## INTRODUCTION

Sequencing microbial cell-free DNA (mcfDNA) in plasma represents integration of progress in genomic sequencing, computation capacity, and recognition of cell-free DNA as a clinically useful blood analyte (1–3). Over the last 4 decades, PCR-based tests, specifically multiplexed broad syndromic panels, have made welcomed contributions to infectious disease diagnostics but fall short of desired performance, including breadth of pathogen detection, and require samples of infected tissue or body fluid (4). Broad-range PCR testing arguably facilitates considering a wider range of potential pathogens but is still only limited to bacteria and fungi (5). A recent meta-analysis, which included 20 studies that satisfied the Quality Assessment of Diagnostic Accuracy Studies (6) to assess the diagnostic accuracy of next generation sequencing in distinguishing infectious diseases, concluded that this group of technologies demonstrated satisfactory diagnostic performance for infections and yielded an overall detection rate superior to conventional methods (7). Four of the studies included in this review employed plasma mcfDNA sequencing. Moreover, early experience with plasma mcfDNA sequencing suggests this new approach, especially when applied early in a patient’s clinical course and for specific use cases, has potential to improve upon the above-noted shortcomings (8, 9). Plasma mcfDNA sequencing enables unbiased pathogen detection through noninvasive sampling with rapid turnaround, creating opportunities to enhance diagnosis of bloodstream and deep-seated infections (10–12). These capabilities are most urgently needed among immunocompromised patients who often have the broadest range of pathogens and are most vulnerable to serious consequences from infections. The Karius Test is an analytically and clinically validated mcfDNA sequencing test commercially available for US inpatients since 2017 as a laboratory developed test from Karius, Inc. The test can identify and quantitate molecules/μL (MPM) mcfDNA in plasma for >1,500 bacteria, DNA viruses, fungi, and parasites. The analytical and clinical validation of the test was previously reported (12). Since the time of this study, others have reported how this unbiased test may contribute to the diagnosis and management of life-threatening infections in immunocompromised patients. These contributions include minimizing invasive procedures (13), reducing time to specific etiologic diagnosis of infections compared with standard of care (SOC) microbiological testing (14), and optimizing antimicrobial therapy (15, 16). In contrast, several retrospective, observational reviews of Karius Test utilization concluded that in routine clinical practice the diagnostic and clinical impact of the test was limited, which highlights the need for diagnostic stewardship to optimize implementation and maximize clinical utility in specific patient populations (17–19).

Plasma mcfDNA sequencing for infectious disease diagnosis performance at scale with respect to time to results, laboratory quality metrics, positivity rates, and diversity of taxa detected has not been previously reported. Here, we review the results for a large commercial laboratory testing cohort of over 18,000 plasma samples from over 15,000 patients in a hospital setting with the primary objective to provide additional insights about the breadth and complexity of microbial identifications. While doing so, we also characterize clinical use in a subset of the patients in this test cohort.

## MATERIALS AND METHODS

### Commercial laboratory test cohort.

The Karius Test results for patients from across the United States were evaluated for reported pathogens and patient data (including basic demographics, ordering clinician, and ICD-10 codes, if provided) obtained from the test request forms (TRF) for all samples tested from 1 April 2018 through mid-September 2021. Laboratory quality metrics were gathered for all samples collected from 1 April 2018 through the end of September 2021. Diagnosis codes submitted via TRFs were summarized at both the chapter level and Clinical Classifications Software Refined categories (20, 21). Immunocompromising conditions were then flagged using definitions published by the Agency for Healthcare Research and Quality (22–24).

### The Karius Test.

Plasma mcfDNA sequencing was performed as previously described (12) in the Karius clinical laboratory, certified under the Clinical Laboratory Improvement Amendments of 1988 and accredited by the College of American Pathologists. Briefly, whole-blood samples were collected in either BD Vacutainer plasma preparation tubes (PPTs) or K2-EDTA tubes. After plasma had been separated from cells, the sample was stable at ambient temperature for 96 h and at −20°C for 6 months. Upon receipt at Karius, controls for carry-over, sequencing bias, metagenomic sequencing quality, and sample mix-ups were added to the sample.

In addition, two types of batch controls were run alongside patient samples. Four replicates of environmental control samples containing buffer instead of plasma were processed in parallel with patient samples from accessioning to report generation. Environmental controls were used to monitor microbial DNA signals arising from the background at the time of batch processing. The estimated taxon abundances from the environmental control samples within the batch were combined to parameterize a model of read abundance arising from the environment with variations driven by counting noise. Two assay controls, each containing four distinct species ranging in GC-content from 30% to 65% at high or low concentrations were included in each batch. All four spiked microorganisms and no others had to be detected within a specified MPM range in both assay controls to pass the final quality control inspection.

Proprietary chemistries were used to enrich samples for mcfDNA without preselecting pathogens to test. Automated DNA extraction and sequencing library preparation protocols were optimized for high speed and low pathogen bias. Single-end, 76- or 56-cycle sequencing was performed on NextSeq 500 instruments (Illumina, San Diego, CA) with an average of >20 million reads/sample. Double-unique dual indexes were used to ensure robust sample demultiplexing. Sequencing data were processed using a proprietary analytical pipeline, and microbial reads were aligned to a database comprising >20,000 curated assemblies from >16,000 species of which >1,500 taxa are in the clinical reportable range (CRR) of the test, including bacteria, DNA viruses, fungi, and parasites.

The selection of organisms in the CRR was performed as follows. A candidate list of human pathogens was generated by two board-certified infectious disease physicians by including: (i) DNA viruses, bacteria, mycobacteria, fungi, and parasites from the standard textbooks and other relevant references, (ii) organisms in the pathogen database referenced in published case reports, and (iii) reference genomes sequenced from human clinical isolates with publications supporting potential pathogenicity. Organisms from the above list that were associated with high-quality reference genomes, as determined by our reference database quality control process, were used to further narrow the range. Finally, organisms at risk of generating common false-positive calls because of sporadic environmental contamination were removed from the CRR. The current CRR is available at https://kariusdx.com/the-karius-test/pathogen-list/.

Statistical significance values were computed for each estimated taxon abundance in each nonenvironmental control sample. Those within the CRR at high significance levels above background comprised our candidate detections. Final detections were made after additional filtering was applied, which accounted for read location uniformity, read percent identity, and cross-reactivity originating from higher abundance detections. The microorganism detections that passed these filters were reported along with abundances in MPM, a unique, absolute quantification capability of the Karius Test shown to correlate well with single-analyte qPCR measurements of DNA viruses (25, 26).

The reports also contained median and range of MPM values observed for each microorganism reported in the last 1,000 specimens, as MPM values from different microbes are not comparable, and a reference interval determined from 675 asymptomatic donors for comparison. We routinely analyzed the raw data for mcfDNA from potential pathogens, including those present at levels below our standard laboratory report thresholds. For this study, we focused on microorganisms identified in statistically significant amounts.

Notable improvements in the test wet bench procedures and analytical pipeline, as may be anticipated, occurred during the study period, and are provided in Table S1 in the supplemental material. We used the operational classes of pathogens described by Relman, Falkow, and Ramakrishnan (27). The operational classes include obligate, commensal, zoonotic, and environmental pathogens here to categorize some the pathogens detected.

### Data analytics.

Data analysis and visualization were conducted using Python v3.9.7, pandas v1.3.4, matplotlib v3.4.3, and seaborn v0.11.2 (28–30). Given the taxonomy and nomenclature for some genera continue to evolve, we selected three (*Legionella*, *Nocardia*, and Mycobacterium) to examine species detections, especially multiple species codetections, more closely. All analyses were done by Karius staff.

### Ethical considerations.

Using the Department of Health and Human Services regulations found at 45 CFR 46.104(d)(4), the Advarra IRB determined that this research project was exempt from IRB oversight.

## RESULTS

### Test cohort.

**FIG 1:**
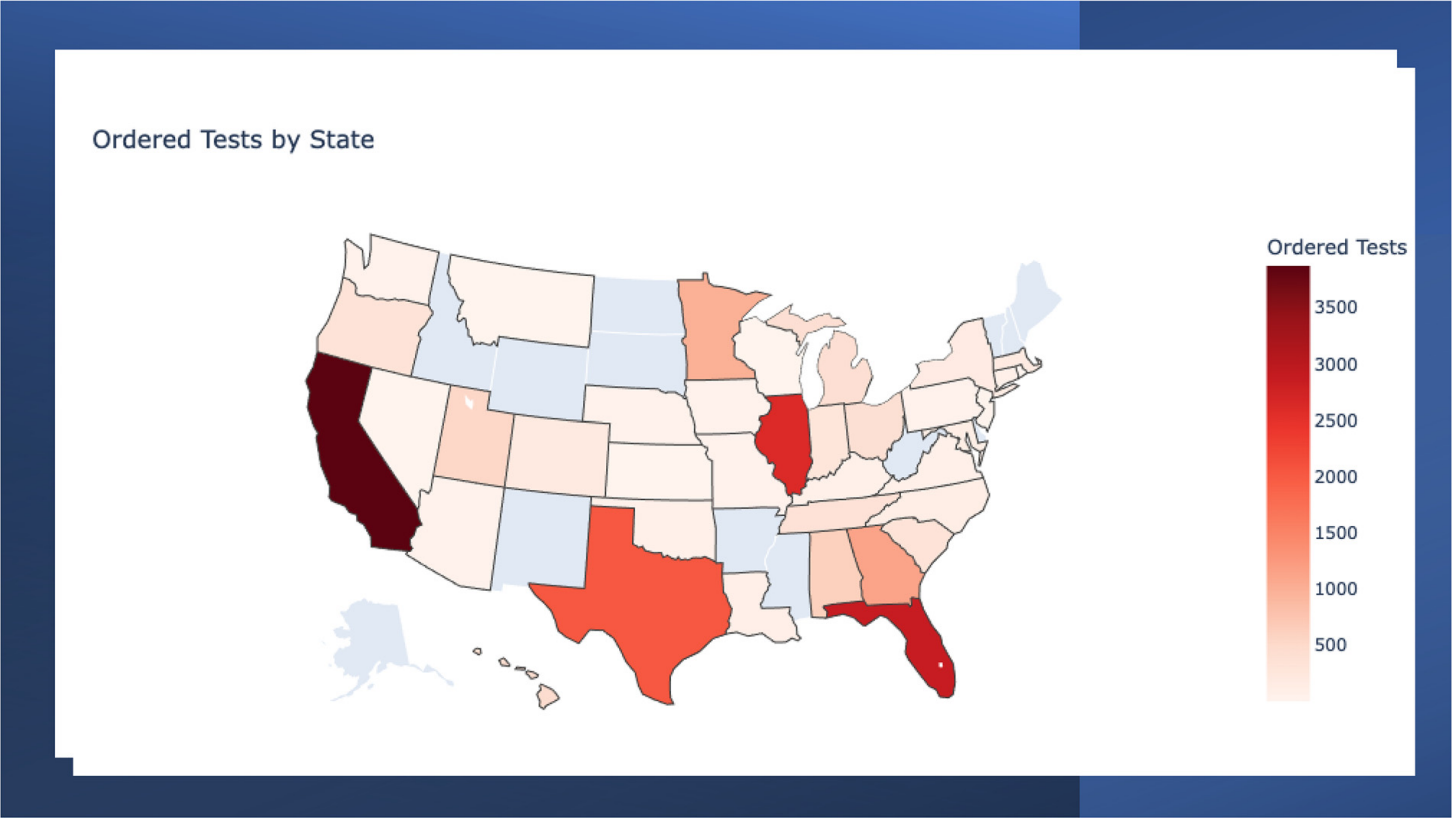
Number of Karius Tests ordered by state from April 2018 to September 2021. No samples were received from the states shaded in blue.

A total of 19,739 samples meeting collection and transport requirements were tested from 16,172 patients in a hospital setting in 39 states and the District of Columbia during the study period. See Fig. 1 for the number of Karius Tests ordered by state. The median time from sample receipt at the Karius laboratory to the delivery of the reported result was 26 h (interquartile range 25 to 28), and the median time from sample collection to the delivery of reported result was 63 h (interquartile range 49 to 89). Ninety-six percent of samples had valid test results. A summary of key quality metrics for the laboratory in this production data set is shown in Table 1. These metrics were not significantly different (two-sided *t* test *P* value >0.01) from those reported for the first 2,000 clinical samples run by the Karius clinical laboratory and reported in the initial validation study (12).

**TABLE 1:** Plasma mcfDNA sequencing clinical laboratory quality metrics in production from April 2018 to the end of September 2021

Metric	Production^*a*^	Validation^*b*^
Sample acceptance rate	98%	98%
Total yield	96%	98%
First pass yield	91%	91%
Results delivered next operational day	90%	90%

a Based on 20,087 clinical samples received during this study.

b Based on 2,000 clinical samples tested as part of the initial Karius Test validation study (12).

Infectious disease and hematology/oncology providers represented most ordering clinicians, 64% (*n* = 9,804) and 14% (*n* = 2,132), respectively, for the 15,424 specimens with a National Provider Identifier indicated. We were able to capture and analyze 18,690 reports from 15,165 patients. Twelve percent (*n* = 1,839) of patients had at least one repeat test during the study interval. Almost two thirds (65%, *n* = 9,798) of patients were adults (i.e., age >18 years). More than a quarter (29%, *n* = 4,423) of patients at the time of diagnostic testing had ICD-10 codes representing a diverse array of clinical scenarios indicated in their TRFs (Table 2). Eighteen percent (*n* = 797) of these patients were indicated as immunocompromised (IC); 717 (16%) had fever; and 230 (5%) had sepsis.

**TABLE 2:** ICD-10 codes by principal diagnosis type for those patients with ICD-10 codes indicated on Karius TRFs (*n =* 4,423) from April 2018 to September 2021^*a*^

Principal diagnosis type (ICD-10-CM chapter)	Total	IC	Fever	Sepsis
(Unmappable)	68			
Conditions not elsewhere classified	1,437		722	
Respiratory system diseases	800			
Neoplasms	580	288		
Infectious/parasitic diseases	543	47		231
Blood diseases	485	320		
Circulatory system diseases	416			
Factors influencing health status and contact with health services	290	140		
Musculoskeletal system diseases	259	9		
Nervous system diseases	242			
Digestive system diseases	162	21		
Skin diseases	111			
Genitourinary system diseases	101	15		
Endocrine/metabolic diseases	91	4		
Injury, poisoning, external causes	83	25		
Congenital malformations	76			
Codes for special purposes	20			
Eye/adnexa diseases	16			
Mental/behavioral disorders	13			
Ear/mastoid diseases	7			
Pregnancy/childbirth	7			
External causes of morbidity	3			
**Total no. of patients by diagnosis chapter**	5,810	869	722	231

a TRF, test report form; IC, immunocompromised. Fever, any ICD-10 starting with “R50”; sepsis, any ICD-10 starting with “A41”; IC, any ICD-10 annotated as immunocompromised from the Agency for Healthcare Research and Quality (AHRQ) code list. Each TRF could contain up to two ICD-10 codes, and each patient had between 1 and 5 unique ICD-10 codes. For the study period, there were 15,165 patients with a positive or negative report (18,690 reports).

### Taxa detection and quantification.

**FIG 2:**
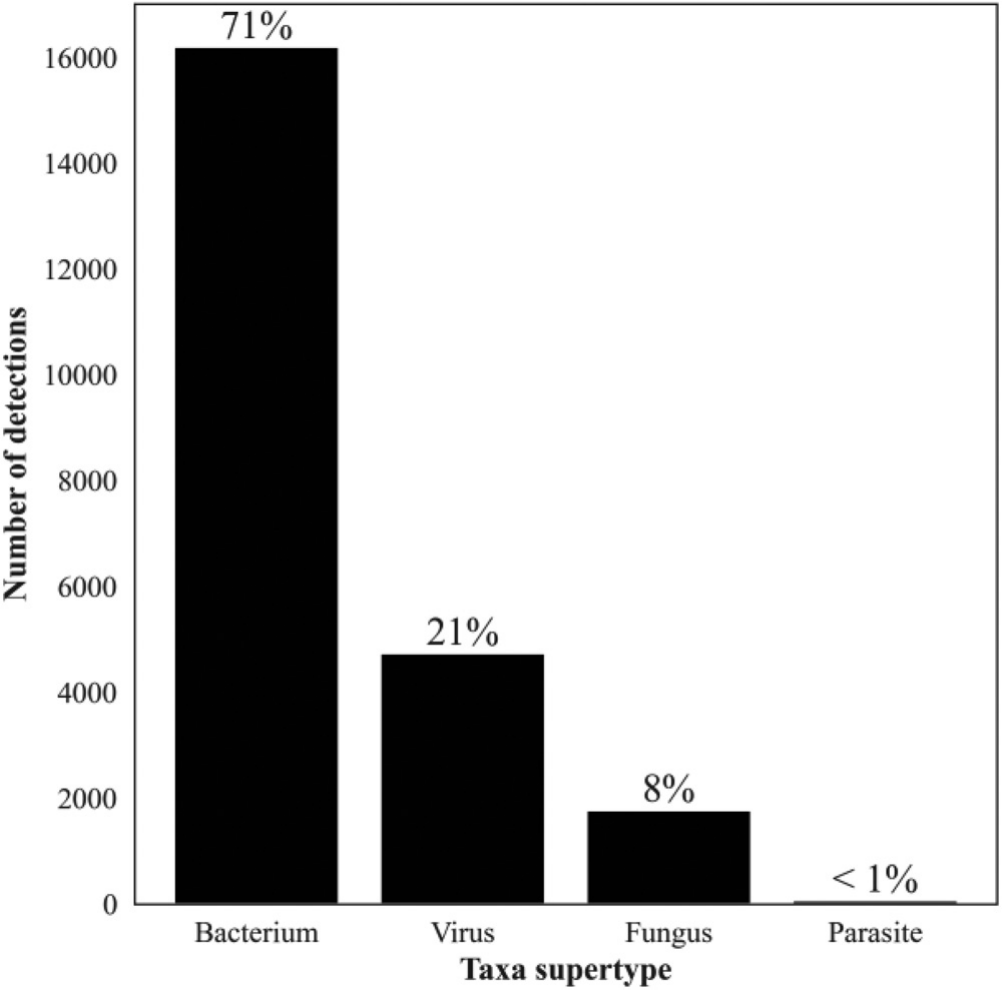
Number of detections by the Karius Test of the different supergroups of taxa from April 2018 to September 2021. *n* = 22,792: bacteria, 16,221; viruses, 4,737; fungi, 1,758; and parasites, 70. Percentages reflect the proportion of total number of detections spanning 701 microbial taxa.

**FIG 3:**
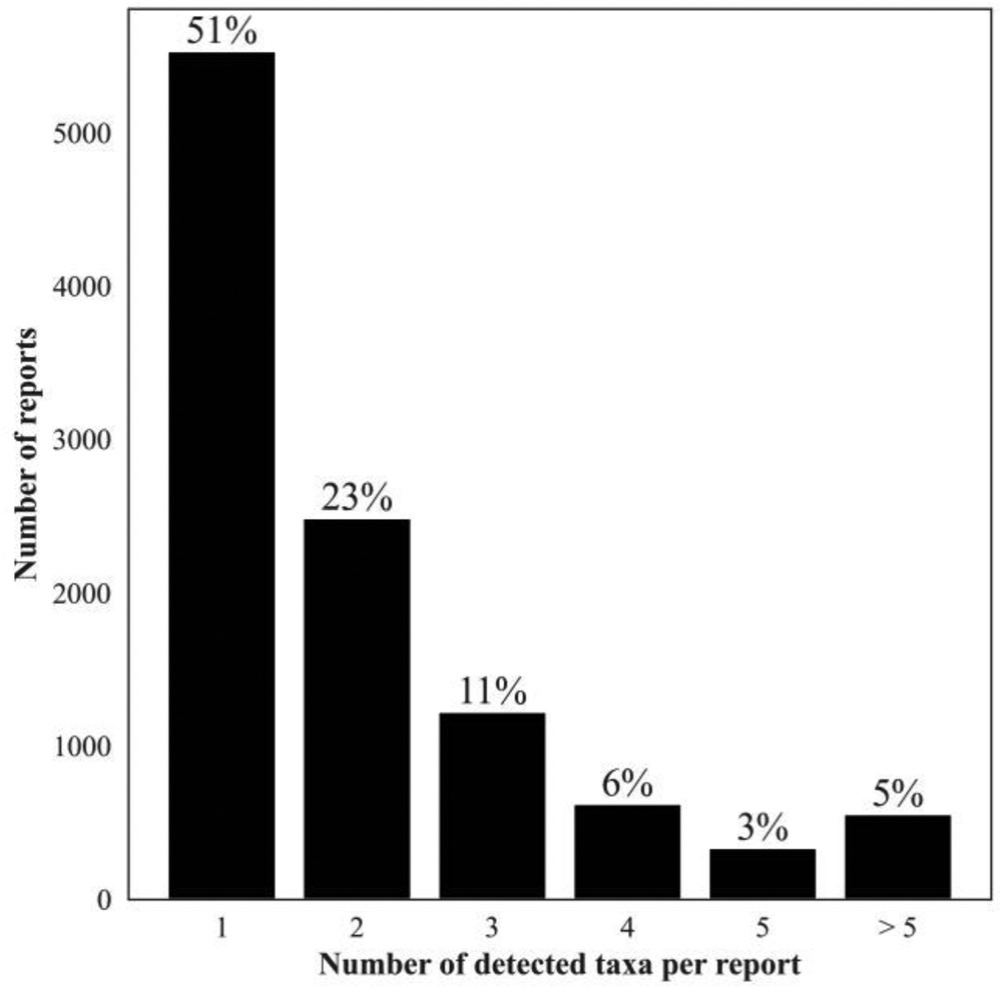
Number of detected taxa counts per report for all positive reports, April 2018 to September 2021. Percentages reflect the proportion of all positive reports (*n* = 10,752).

Of samples yielding a valid result, 7,938 (42%) reported a negative test with no pathogens identified. The remaining 10,752 (58%) Karius Test reports from 8,849 patients had at least one microbe identified (5,531 [30%] only one) representing 701 unique microbial taxa (526 [75%] bacteria, 103 [15%] fungi, 47 [7%] viruses, and 24 [3%] parasites) and a total of 22,792 detections. The overall frequency of detection for each of these groups is shown in Fig. 2, and the number of detected taxa counts per report for all positive reports is shown in Fig. 3. All the quality control metrics were met for taxa quantification in MPM for 9,690 (90%) samples with positive results. A complete list of all taxa reported along with their frequency of detection and median and IQR for the MPM values are shown in Table S2.

### Top 50 reported taxa.

The top 50 reported taxa and the median, range, and IQR of MPM for each taxon are shown in Table 3. They included 36 bacteria, 9 viruses, and 5 fungi. Together the top 50 taxa included a broad range of commensal and environmental pathogens and represented 15,692 detections (69% of all detections).

**TABLE 3:** Top 50 taxa detected from April 2018 to September 2021

Taxon name^*a*^	No. detected	Median (MPM)	IQR (MPM)
Bacteria			
Acinetobacter haemolyticus	199	142.97	192.68
Bacteroides fragilis	217	264.74	1,046.15
Bacteroides ovatus	133	141.43	476.14
Bacteroides thetaiotaomicron	146	221.79	742.60
Bacteroides vulgatus	392	153.01	413.32
Enterobacter cloacae *complex*	343	821.34	6,742.30
Enterococcus faecalis	582	617.90	3,285.15
Enterococcus faecium	510	687.63	3,958.33
Escherichia coli	1088	684.92	3,623.17
Fusobacterium nucleatum	299	460.25	1,922.08
Haemophilus influenzae	288	157.09	425.59
Haemophilus parainfluenzae	178	284.92	1,121.19
Helicobacter pylori	207	217.44	601.83
Klebsiella pneumoniae	501	910.19	5,889.84
Lactobacillus fermentum	165	256.13	1,058.08
Lactobacillus rhamnosus	281	344.33	1,250.14
Prevotella melaninogenica	509	208.52	545.66
Prevotella oris	170	517.78	2,094.07
Pseudomonas aeruginosa	817	1,005.18	6,874.18
Rothia dentocariosa	110	117.02	256.72
Rothia mucilaginosa	454	177.53	369.36
Serratia marcescens	99	492.72	2,956.54
Staphylococcus aureus	561	838.21	5,665.43
Staphylococcus epidermidis	716	579.58	2,689.68
Staphylococcus haemolyticus	92	1,137.08	5,450.01
Stenotrophomonas maltophilia	169	1,983.18	11,864.14
Streptococcus intermedius	100	354.95	1,048.47
Streptococcus mitis	356	226.15	897.48
Streptococcus oralis	140	235.54	730.42
Streptococcus parasanguinis	186	152.35	298.03
Streptococcus pneumoniae	214	975.48	11,933.10
Streptococcus pyogenes	95	1,064.08	9,773.54
Streptococcus salivarius	104	253.73	799.10
Streptococcus thermophilus	184	190.84	339.33
Veillonella dispar	162	140.88	269.85
Veillonella parvula	256	202.30	476.57
Fungi			
Aspergillus fumigatus	186	300.10	723.60
Candida albicans	260	624.63	2,074.97
Candida glabrata	113	611.85	1,449.20
Candida tropicalis	103	784.49	3,997.85
Pneumocystis *jiroveci*	258	2,452.78	18,084.89
Viruses			
BK polyomavirus	354	472.38	2,677.18
Cytomegalovirus	1275	633.93	3,889.67
Epstein-Barr virus	902	272.34	852.32
Herpes simplex virus type 1	479	754.78	3,208.06
Human herpesvirus 6B	468	518.74	3,268.72
Human herpesvirus 7	98	82.74	342.37
Human adenovirus B	100	3,184.75	69,714.71
Human adenovirus C	171	573.87	10,160.48
Torque teno virus	135	484.19	1,935.97

a Reported taxa reflect those names used in the clinical reportable range of microbes at the time that the tests were performed.

The distribution of these taxa is as follows. There were 11,023 detections of bacteria, including 11 anaerobes (2,730, 25%), 8 Streptococcus spp. (1,379, 12%), 4 *Enterobacterales* (2,031, 18%), 3 Staphylococcus spp. (1,369, 12%)., 2 *Rothia* spp. (564, 5%), 2 Haemophilus spp. (466, 4%), 2 *Enterococcus* spp. (1,092, 10%), and 1 each of Acinetobacter haemolyticus (199, 2%), Pseudomonas aeruginosa (817, 7%), Stenotrophomonas maltophilia (169, 1%), and Helicobacter pylori (207, 2%). There were 3,982 viral detections of 9 different viruses that included 1,275 (32%) cytomegalovirus, 902 (23%) Epstein-Barr virus, 479 (12%) herpes simplex virus 1, 468 (12%) human herpesvirus 6B, 354 (9%) BK polyomavirus, 171 (4%) human adenovirus C, 135 (3%) torque teno virus (TTV), 100 (3%) human adenovirus B, and 98 (3%) human herpesvirus 7. Finally, there were 920 detections of fungi comprising 260 (28%) Candida albicans, 258 (28%) Pneumocystis jirovecii, 186 (20%) Aspergillus fumigatus, 113 (12%) Candida glabrata, and 103 (11%) Candida tropicalis.

### Difficult-to-diagnose uncommon pathogens.

The SOC methods for the organisms listed below have considerable shortcomings, including, but not limited to, sensitivity and specificity, inclusivity, accuracy, time to result, and/or local availability.

**FIG 4:**
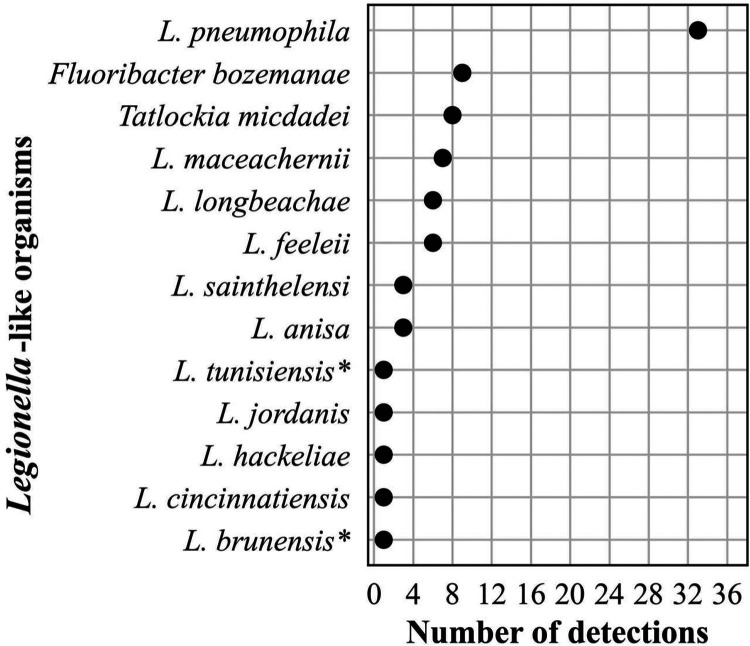
Frequency distribution of *Legionella*-like organisms detected, *n* = 80 (<1% of all bacterial detections, *n* = 16,203), April 2018 to September 2021. *indicates species not associated with human infections.

**Bacteria.** The frequency distribution of the number of detections of *Legionella*-like organisms (*n* = 80) is shown in Fig. 4. Forty-one percent of these detections were the most recognized pathogen, L. pneumophila. Two reports contained codetections of two different species (*L. brunensis*, 400 MPM and *L. hackeliae*, 270 MPM; and *L. feeleii*, 78,508 MPM and *L. tunisiensis*, 76,445 MPM, respectively). Neither *L. brunensis* nor *L. tunisiensis* has been associated with human disease (https://specialpathogenslab.com/legionella-species/), and the MPM values for the codetections in each report were similar.

**FIG 5:**
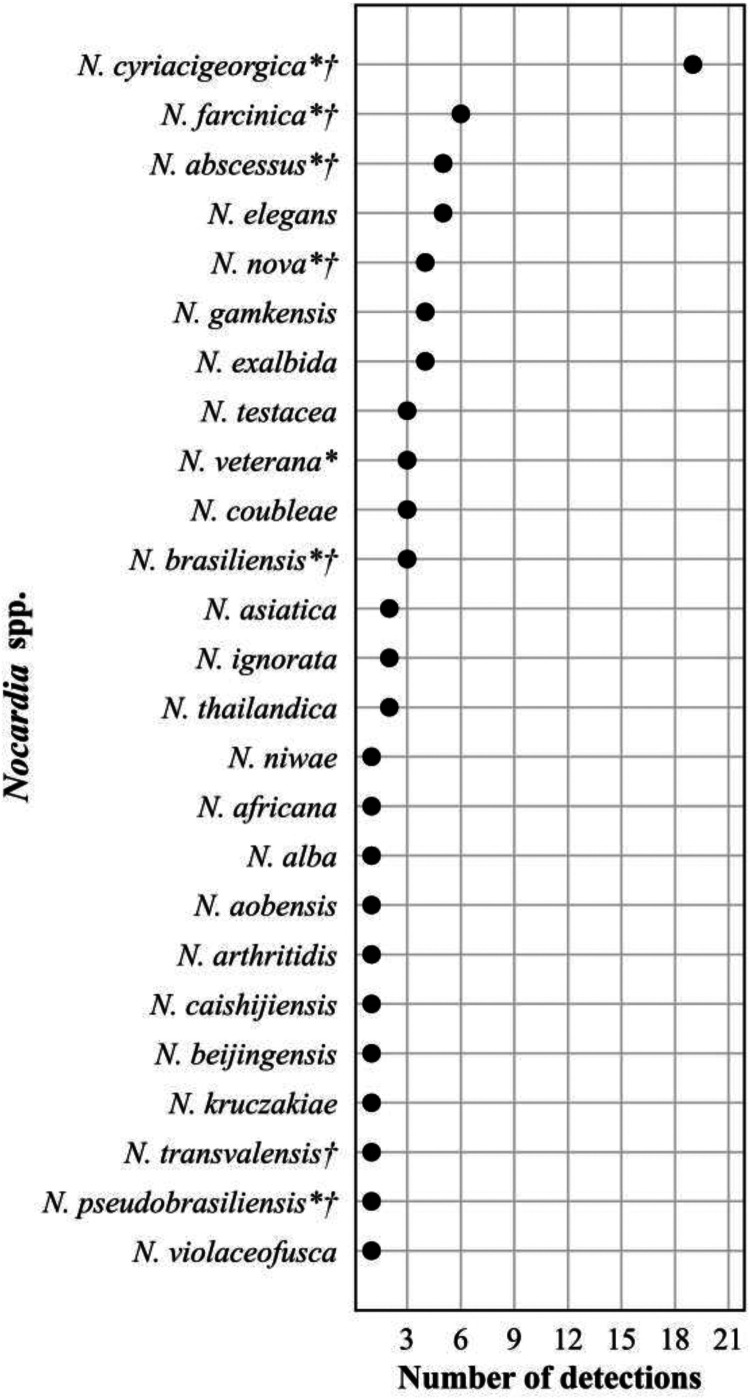
Frequency distribution of *Nocardia* spp. detections, *n* = 76 (<1% bacterial detections, *n* = 16,203), April 2018 to September 2021. An asterisk (*) indicates species more frequently isolated from clinical specimens (69). A dagger (†) indicates species-specific susceptibility patterns (51).

The frequency distribution of *Nocardia* spp. detections, *n* = 76, is shown in Fig. 5. Plasma mcfDNA sequencing detected 25 of the approximately 100 validly named species. Of the 8 species reported to be isolated more frequently from patients, 7 were detected. One species, *N. cyriacigeorgica*, dominated with 19 (25%) detections. Of the 69 patient reports represented by the *Nocardia* spp. detections, 12 (17%) reported ≥2 concurrent species (range 2 to 5). All the codetections were reported with similar MPM values, and 8 (67%) were codetections of closely related species (4 *N. exalbida*/*gam*kensis, 3 *N. elegans*/*nova*/*africana*, 1 *N. kruczakiae/violaceofusca/aobensis*) previously reported to be indistinguishable by mcfDNA sequencing (31, 32).

**FIG 6:**
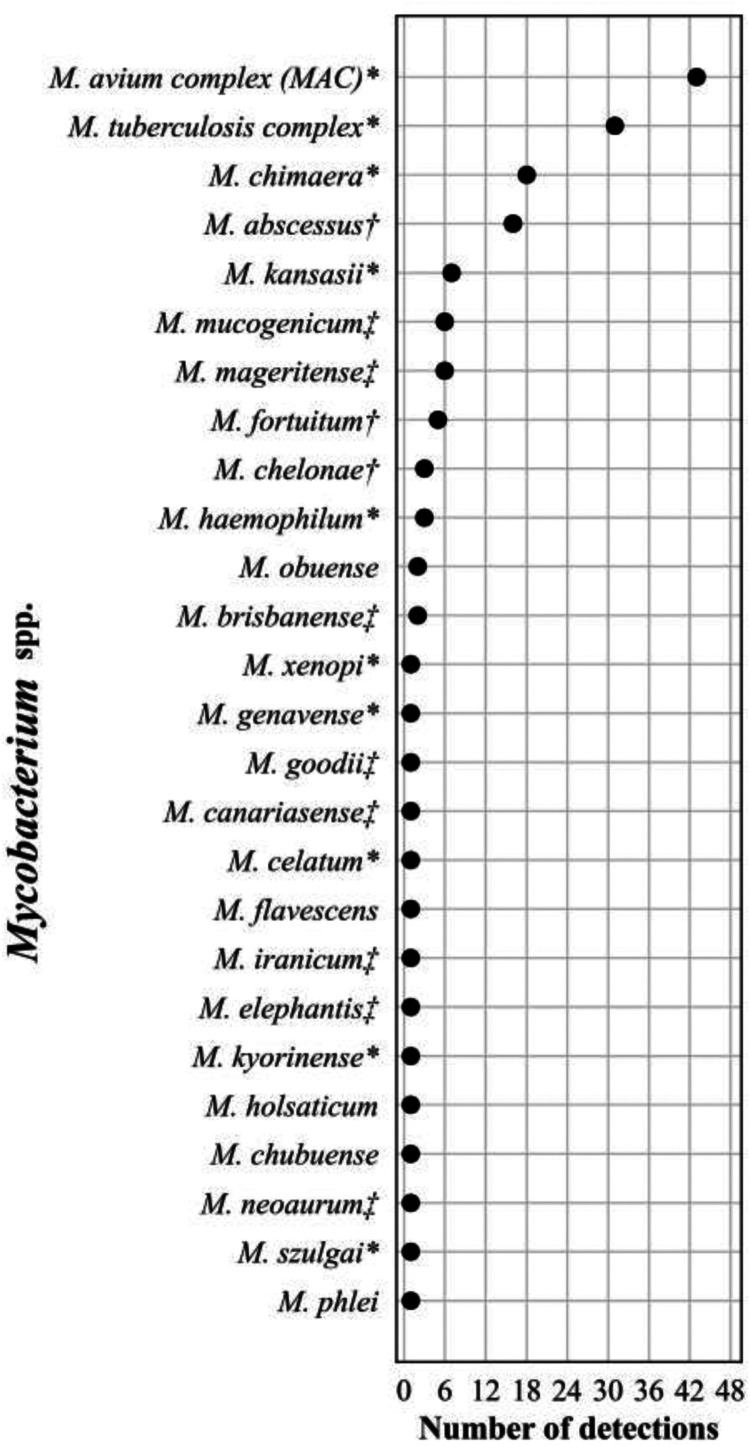
Frequency distribution of Mycobacterium spp. detections, *n* = 156 (1% of all bacterial detections, *n* = 16,203), April 2018 to September 2021. Asterisk (*) indicates slowly growing mycobacteria of established clinical significance (70). Dagger (†) indicates rapidly growing mycobacteria considered common human pathogens. Double dagger (‡) indicates rapidly growing mycobacteria considered less common or rare human pathogens (71).

The frequency distribution of the 156 Mycobacterium spp. detections is shown in Fig. 6. Plasma mcfDNA sequencing detected 107 (69%) slowly growing mycobacteria (SGM) and 49 (31%) rapidly growing mycobacteria (RGM). The frequency of species distributions of these three genera showed similarities in that several species were predominant, followed by long tails of uncommon to single-species detections. We reported ≥2 concurrent species (range 2 to 6) in 6 (4%) of the 144 reports, including Mycobacterium spp. All the codetections were reported with similar MPM values. Three of the reports contained M. avium *complex*/*chimera* and one each M. avium complex/*celatum*/*kyorinense*, *M. brisbanense*/*mucogenicum*/*obuense*, and *M. chubuense*/*elephantis*/*flavescens*/*goodii*/*holsaticum*/*phlei*.

The codetections of multiple *Legionella*, *Nocardia*, and Mycobacterium spp., respectively, within the same sample and their resolution are shown in Table S3. We contrasted the pattern we would expect to see in the alignments in three hypothetical scenarios: (i) a true coinfection of two or more species in the Karius database, (ii) a single species in the Karius database, and (iii) a single species not in the Karius database. In each scenario, we expected certain relative proportions of reads aligning uniquely to each species in the database: those shared among closely related species, those shared among distantly related species, and those aligning more broadly across the entire genus or family. We also considered the BLAST percent identity of the alignments for each scenario and subset of reads. In 19 cases, a single species not in the Karius database seemed to fit best, while the remaining case represented a true codetection of 3 different mycobacteria species (*M. brisbanense*, M. mucogenicum, *and M. obuense*) (data not shown).

**FIG 7:**
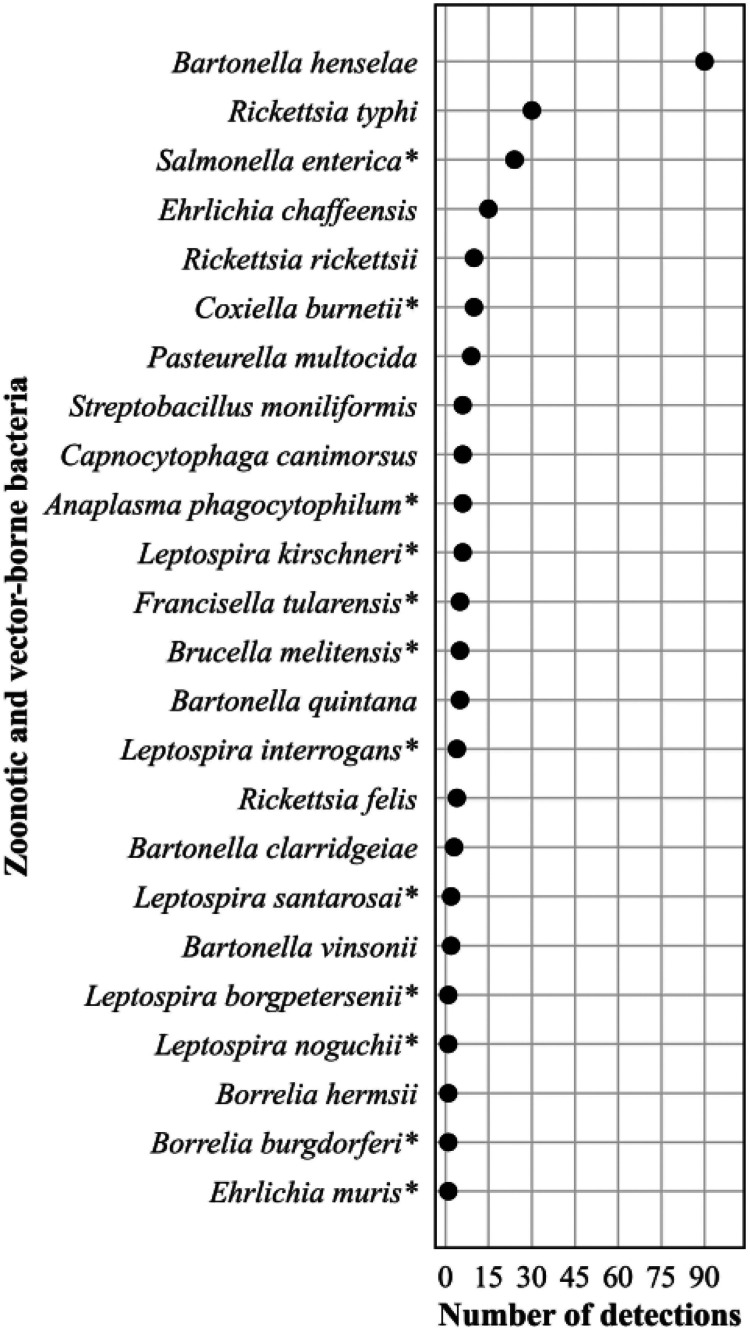
Frequency distribution of zoonotic and vector-borne bacteria detections, *n* = 247 (2% of all bacterial detections, *n* = 16,203), April 2018 to September 2021. Asterisk (*) indicates bacteria causing a nationally notifiable infectious disease (72).

The frequency distribution of the 247 (3% of all bacterial detections) zoonotic and vector-borne bacterial detections are shown in Fig. 7. Bartonella henselae predominated with 90 (36%) detections.

### Fungi.

**FIG 8:**
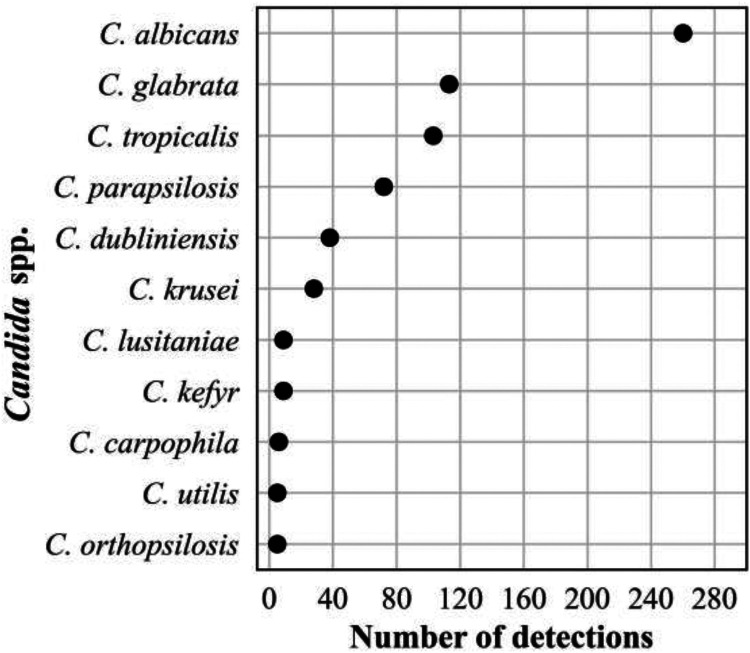
Frequency distribution of *Candida* spp. detections, *n* = 648 (36% of all fungal detections, *n* = 1,776), April 2018 to September 2021.

**FIG 9:**
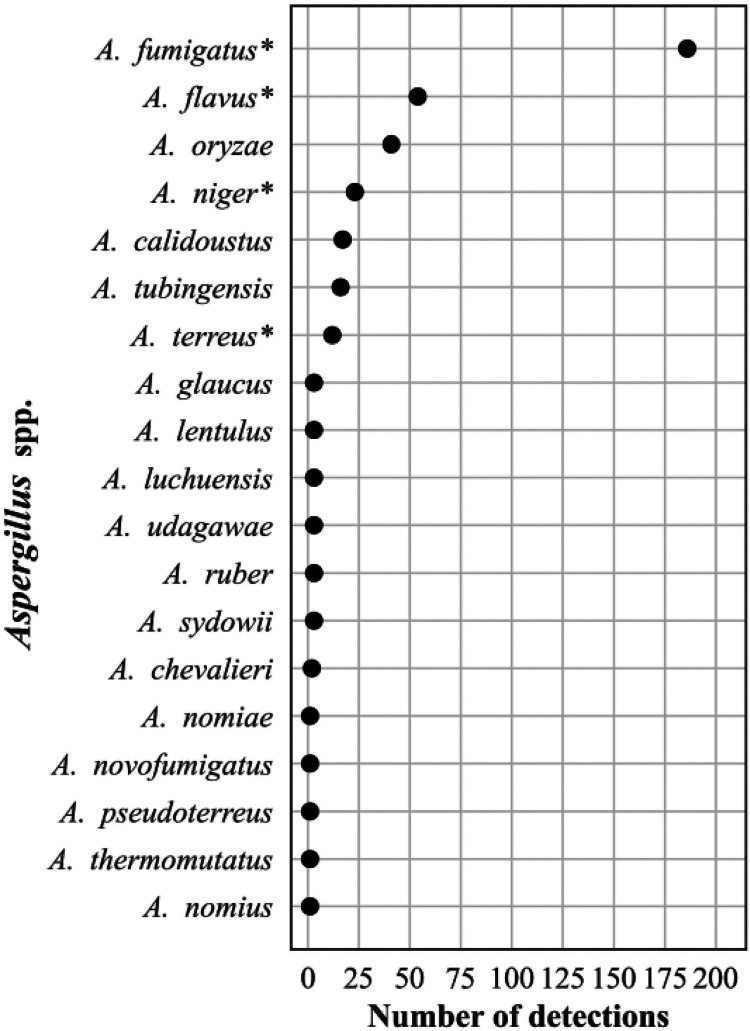
Frequency distribution of Aspergillus spp. detections, *n* = 374 (21% of all fungal detections, *n* = 1,776), April 2018 to September 2021. Asterisk (*) indicates most common pathogenic species (73).

**FIG 10:**
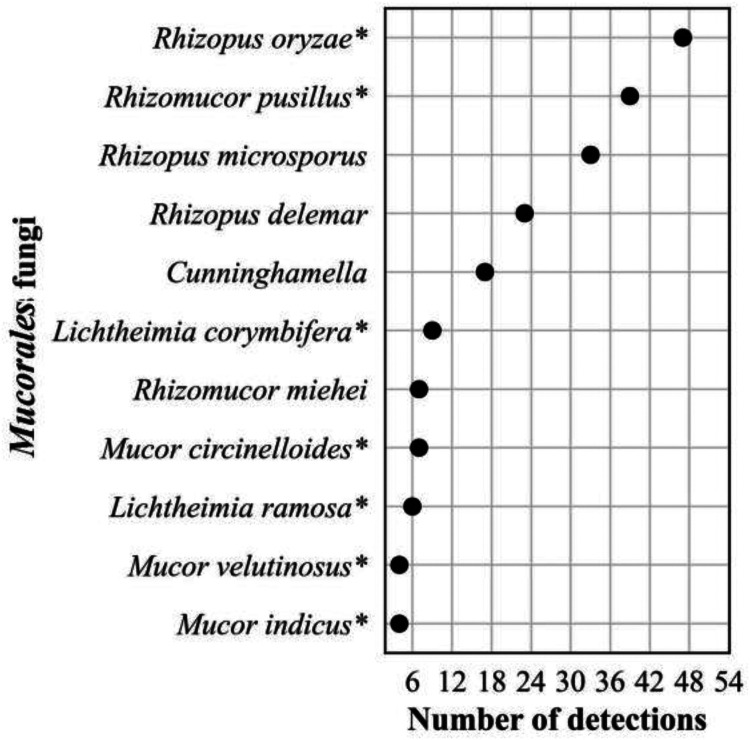
Frequency distribution of detections in the order *Mucorales*, *n* = 196 (11% of all fungal detections, *n* = 1,776), April 2018 to September 2021. Asterisk (*) indicates taxa implicated in human mucormycosis (74).

**FIG 11:**
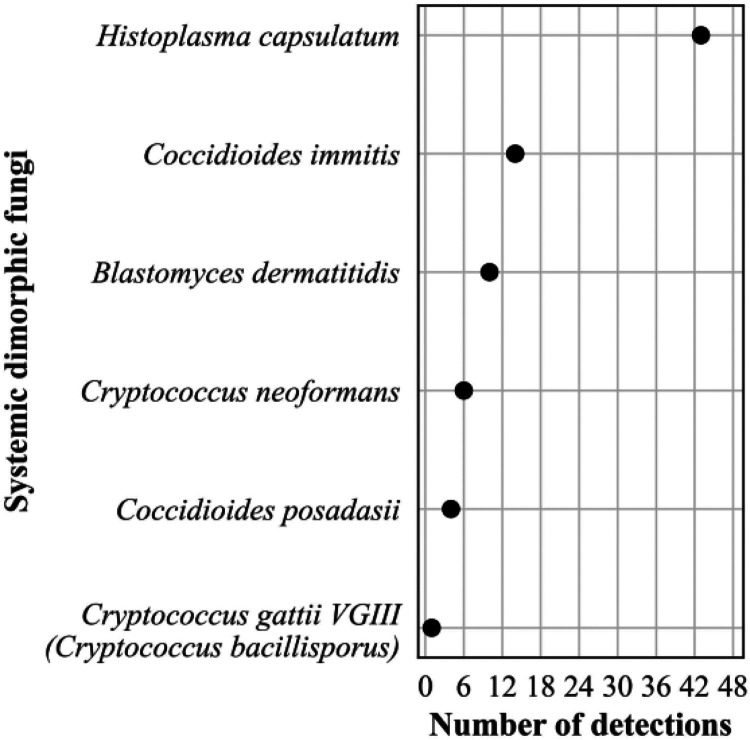
Frequency distribution of detections of systemic dimorphic fungi, *n* = 78 (4% of all fungal detections, *n* = 1,776), April 2018 to September 2021.

**FIG 12:**
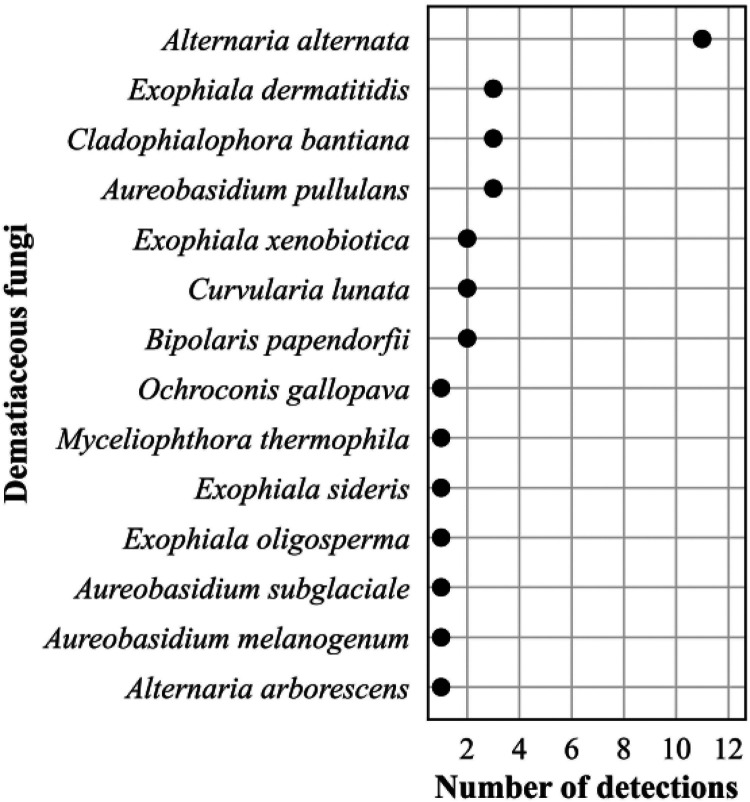
Frequency distribution of detections of dematiaceous fungi, *n* = 33 (2% of all fungal detections, *n* = 1,776), April 2018 to September 2021.

The frequency distribution of the 632 *Candida* spp. detections is shown in Fig. 8; 374 Aspergillus spp. detections in Fig. 9; 196 detections in the order *Mucorales* in Fig. 10; 78 detections of the systemic dimorphic fungi in Fig. 11; and 33 detections of dematiaceous fungi in Fig. 12. We detected 9 microsporidia, including 5 Enterocytozoon bieneusi and one each of E. cuniculi, *E. hellem*, *Anncaliia algerae*, and *Vittaforma corneae*. In addition, Pneumocystis jirovecii (258 detections) was among the top 50 taxa detected.

### Eukaryotic parasites.

**FIG 13:**
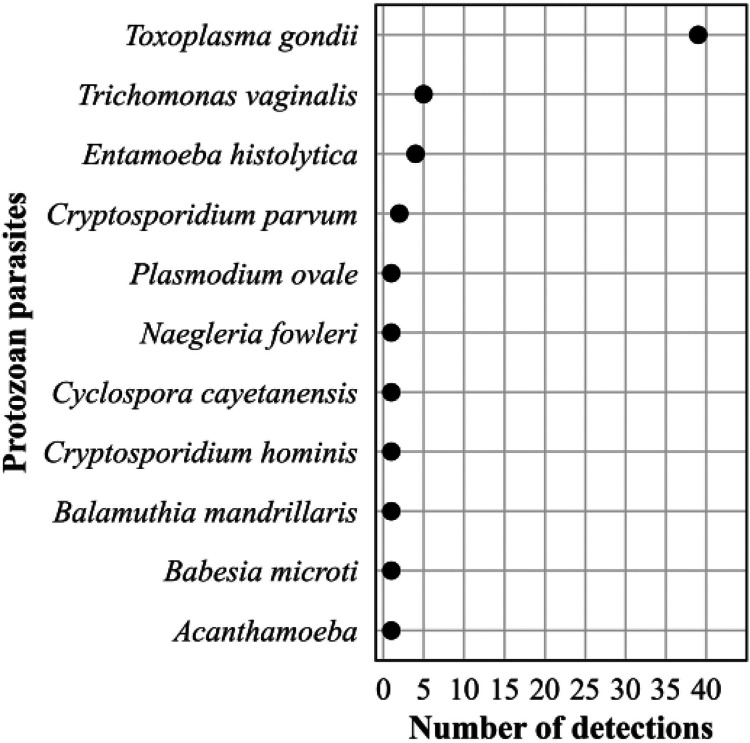
Frequency distribution of detections of protozoan parasites, *n* = 57 (89% of all eukaryotic parasite detections, *n* = 64), April 2018 to September 2021.

The frequency distribution of the 57 (89% of 64 parasite detections) protozoa is shown in Fig. 13. Among the protozoan parasite detections, 68% were Toxoplasma gondii, and 14% were pathogenic amoebae. Among the 7 (11%) helminthic parasites, we detected 4 nematodes (all Strongyloides stercoralis), 2 cestodes (both Echinococcus multilocularis), and 1 trematode (Schistosoma mansoni).

## DISCUSSION

We report the largest testing cohort of patients in which mcfDNA was identified and quantified. The key clinical laboratory quality metrics in this large cohort mirror what was reported with a much smaller cohort in the initial validation study (10), demonstrating that the Karius Test is robust and can be performed at scale in a clinically relevant time frame. These mcfDNA sequencing data reaffirm the ubiquity of some infections, as commonly expected microbes were detected in most patients while less common microbes were rarely detected. However, notably, the unbiased approach of the plasma mcfDNA sequencing made those “rare” detections possible, whereas conventional diagnostics require targeting specific organisms. Identifying optimal utilization, both clinical indications and timing, of the plasma mcfDNA sequencing in future studies to augment clinical decision-making and integration into current testing algorithms (17, 18, 33–35) will serve to improve the utility of the test. Examples of such studies include two recently completed prospective, observational clinical trials of the use of the Karius Test to diagnose pneumonia in immunocompromised patients (NCT04047719) (36) and infections in stem cell transplant inpatients and outpatients over time (NCT02804464) (26), respectively.

Our finding that 58% of the Karius Test reports identified ≥1 pathogen is substantially lower than the 70 to 85% reported for the test when applied to well-defined clinical uses (13, 14, 37). These findings support the need for diagnostic stewardship and additional clinical studies demonstrating how yield differs and should be carefully interpreted according to the specific patient population and disease prevalence considered.

The Karius Test provides absolute quantification of reported microbes in MPM. Generally higher concentrations have been found in confirmed infections, but since considerable overlap of MPM values exists with unlikely infections and with the asymptomatic cohort, threshold values for confirmed infections have not been established (12). However, following the decay of mcfDNA quantitatively by serial testing may have important implications for individual patient management in assessing the effectiveness of antimicrobial therapy and other medical or surgical interventions (8, 38, 39), but needs further study. MPM values can be influenced by several factors specific to the microbial genome such as turnover rate and genome size (12). Confounding patient variables (e.g., infection site, therapeutic interventions, and immune status) may also influence this measure. Lack of the clinical context prevented analyzing the data from those patients who had repeated testing to determine whether these tests were performed serially to diagnose new suspected infections, monitor therapy response, or conduct pathogen surveillance among immunocompromised patients as suggested by others in previous studies. Even with the clinical context, the data would not shed light on reproducibility of the test since plasma mcfDNA has a short half-life (16 min to 2 h) and may increase as infection progresses or decreases in response to therapeutic interventions. The precision (reproducibility) of mcfDNA sequencing was assessed in the analytical validation by testing 13 representative infections in duplicate on each of 20 days; the within run and between run coefficients of variation for MPM values were 18.2% and 19.2%, respectively (12).

Notable among the most common detections were the many commensal bacterial and fungal pathogens that cause serious and often invasive infections in patients with relevant risk factors (e.g., immunocompromised). Among the DNA viruses most detected by the Karius Test (e.g., *Herpesviridae*), all could represent latent infection, reactivation, or active infections depending on the respective clinical conditions; regardless, the detection of these viruses may be of particular concern for immunocompromised patients for whom they may cause considerable morbidity (40). TTV has a remarkable ability to produce chronic infections with no clearly associated clinical manifestations, so they are generally considered orphan viruses. However, detection and quantification of TTV in plasma have proven clinically useful in assessment of the kinetics of functional immune competence in a variety of solid organ transplant patients (41). Considering TTV accounts for about 70% of the total human plasma virome, their detection among the top viruses detected is not surprising. A limitation of the Karius Test is that it does not detect RNA viruses.

A key benefit of unbiased mcfDNA sequencing identified in this study is the ability to detect diagnostically challenging microbes such as opportunistic and systemic dimorphic fungi and zoonotic and vector-borne pathogens (42). These pathogens often carry a sense of urgency for the management of the individual patient and even for the public’s health, as they may be associated with considerable morbidity and potential mortality. As some are uncommonly expected pathogens, they present a major challenge for clinicians in considering and ordering appropriate SOC testing to capture all possible pathogens. For the laboratories, the microbiologic diagnosis of these infections often represents a major challenge for SOC methods, as has been described by others (43, 44).

Plasma mcfDNA sequencing has the potential to enhance disease surveillance, as illustrated by the systemic dimorphic fungi detections. At 43 detections (55% of dimorphic fungal identifications), Histoplasma capsulatum predominated, very likely related to its wide geographic range and opportunities for environmental exposure and mirrors what is known about the epidemiology and incidence of systemic dimorphic fungal infections (45). The relative frequency of detections of *Coccidioides immitis* and *posadasii* and Blastomyces dermatitidis may have been influenced by the geographic bias in this study sample cohort. Of the nine Cryptococcus spp. detected, *gattii* was detected only once compared with *neoformans*, reflecting its restricted geographic distribution and the overall rarity of infections in the United States (46).

Among the rarely detected microbes during the study period are some deserving further mention. The detection of *Legionella*, an obligate pathogen, signals public health concern, whether community or hospital acquired. L. pneumophila serogroup 1 (LP1) is estimated to cause 84% of community acquired Legionnaires’ disease (LD) (47); however, other serogroups of L. pneumophila and other *Legionella* spp. may cause 60% of hospital-acquired LD (48). Urinary antigen detection is the most common diagnostic in the United States and Europe; yet, given its specificity for LP1, as many as 40 to 50% of patients with non-LP1 legionellosis could be missed (49, 50). Plasma mcfDNA sequencing provides comprehensive testing for *Legionella* spp. and *Legionella*-like organisms in one diagnostic test and could thereby expand the known LD epidemiology, particularly in nosocomial cases.

*Nocardia* has 8 species-specific drug susceptibility patterns (51). While accurate species identification can predict antimicrobial susceptibility patterns, molecular methods are required for accurate identifications but are not widely available. The Karius Test detected in our cohort 7 of the 8 species with recognized susceptibility patterns and can provide results more rapidly than existing approaches to species identification.

Finally, culture for mycobacteria, while a complicated and lengthy process, is considered the gold standard, supplemented by direct detection of M. tuberculosis complex by nucleic acid amplification tests (NAATs) in many laboratories. However, NAATs for the direct detection of nontuberculous mycobacteria are not widely available, and accurate identification to species level from cultured isolates remains challenging for most laboratories. The *M. chimaera* may be overrepresented in our cohort since the Karius Test was optimized for its detection following reports of infections occurring after surgeries employing contaminated cardiopulmonary bypass devices (52). However, these detections demonstrate the capability of mcfDNA sequencing to provide comprehensive identification of these important obligate and commensal pathogens directly from plasma and provides additive diagnostic value to the above-mentioned SOC methods (53).

Plasma mcfDNA sequencing offers a noninvasive means of detecting microbial infection and capturing species diversity, potentially revealing new insights on genetic complexity not resolved by current taxonomic classification. Still, our findings highlight the need to expand currently available reference genomes, as many species across various genera remain undiscovered or undescribed (54, 55). The species codetections demonstrated among the genera we highlighted, *Legionella*, *Nocardia*, and *Mycobacteria*, were resolved as a single species not in the Karius database in all but one case, which was resolved as a true codetection of 3 different *Mycobacteria* species. We estimate that codetections of genetically similar microbes as described above occurred in ≤3% of all detections in the CRR. Similar challenges exist for broad-range PCR testing (5, 56) and occurred with adoption of proteomic identification by MALDI-TOF mass spectrometry (57), but the scope of the problem occurs less with next-generation sequencing (NGS) approaches.

This study has several limitations preventing us from directly comparing Karius with orthogonal SOC microbiological test results and fully elucidating its impact on patient care. While infectious disease physicians at Karius routinely communicated with ordering clinical providers regarding microbes identified as pathogens of special clinical significance (e.g., rare, and diagnostically challenging), any data collected were for the purposes of providing test interpretation support and facilitating in-house testing improvements. Karius did not have data use agreements with the over 200 referring institutions for publication purposes. However, numerous peer-reviewed publications have reported positive and negative percent agreement of plasma mcfDNA sequencing results with SOC microbiology results in identifying clinically adjudicated causes of infection across a broad range of patient populations and microbes. These studies have reported accuracy assessments on a microbe-by-microbe basis and have gone even further to report clinical validity as well. Six representative studies including 683 patients are summarized in Table S4 (8, 11, 12, 14, 58, 59).

In the studies above, the positive percent agreement (PPA) compared with SOC methods were similarly high (≥87%), but perhaps more importantly, plasma mcfDNA sequencing had higher diagnostic yields than SOC tests for diagnosis of the adjudicated causes of infections. The analytical sensitivity and breadth of microorganisms detected, combined with the diversity of the microbes across patients does present challenges in achieving high diagnostic specificity or NPA range (52.3 to 100%) compared with SOC methods. Even so, the initial analytical validation demonstrated very low levels of false reporting of mcfDNA not in the original plasma sample, consistent with high reproducibility of mcfDNA detection across independent runs (12). All samples tested in these previous publications were analyzed in the same laboratory using fundamentally the same wet bench procedures and computational algorithm as the samples reported here. Consequently, we are confident that the performance metrics in our test cohort are comparable with what has been previously reported.

The clinical context for using the Karius Test was provided in only 29% of patients and is therefore limited and likely biased as that information was voluntarily provided, may have been incomplete, and would reflect the clinician’s perspective at the time they ordered the test versus the final diagnosis. However, others have reported increased diagnostic yield of plasma mcfDNA sequencing compared with SOC tests in certain clinical scenarios (14–16, 60). Some have noted the test to be commonly applied in managing severely ill, especially IC, patients (61) who are more likely to be infected with unusual and pathogens that are difficult to diagnose (62, 63). The patient populations and disease states in these studies reflect this cohort’s most associated ICD-10 codes, thus providing insight about the real-world use of the test. In addition, infectious disease and hematology/oncology providers comprised most (78%) of the ordering clinicians, aligning with expectations that these clinicians commonly care for critically ill and IC patients with difficult-to-diagnose infections. Constraints of the underlying data structure prevented analyzing the data from the 12% of patients who had repeated testing to determine whether these tests were performed serially to diagnose new suspected infections or to monitor therapy response or conduct pathogen surveillance among immunocompromised patients as described by others (8, 26, 64–66). Further, the underlying data structure limited our ability to stratify the results by improvements to the bioinformatics pipeline over the course of the study period. However, the criteria for deciding whether a taxon was reported have only changed marginally during this study period.

Unbiased plasma mcfDNA sequencing can potentially enhance patient outcomes by direct and timely recognition of pathogens in specific clinical scenarios as well as benefit the overall public health by increasing our understanding of the epidemiology of emerging infectious diseases such as monkeypox virus (M.S. Lindner, K. Brick, N. Noll, S.Y. Park, et al., submitted for publication) or Borrelia miyamotoi (67). Further, this powerful, novel diagnostic tool may facilitate medical advances through recognizing previously unanticipated pathogens, as noted when mcfDNA sequencing was leveraged in a research-use-only modality to identify porcine cytomegalovirus infection in a patient who had received a genetically modified porcine-to-human cardiac transplant (68). As with any advanced diagnostic tool, careful, timely clinical application with expert guidance and interpretation of its results as well as appropriate diagnostic stewardship will optimize its application to offer even greater clinical impact. In addition, the development of robust clinical outcomes studies to evaluate the clinical impact and cost effectiveness of plasma mcfDNA sequencing for specific clinical indications to guide use remains a top diagnostic stewardship priority. Leading test use clinical indications reported in the literature include pneumonia in immunocompromised patients, fever of unknown origin, febrile neutropenia, invasive fungal infections, endocarditis, and complicated community acquired pneumonia. Also, the breath of pathogens detected in this study suggest mcfDNA sequencing may be useful as a single test for comprehensive diagnosis of zoonotic and vector-borne infections, systemic dimorphic fungal infections, legionellosis, and infections caused by other diagnostically challenging microbes.

This is the largest reported cohort of patients tested using plasma mcfDNA sequencing. As such, it represents the first report of a clinical grade metagenomic test performed at scale, with laboratory quality metrics that mirror what was reported in the initial validation study and demonstrates that plasma mcfDNA sequencing is robust and can be performed in a clinically relevant time frame. The large number of samples included also provides new insights into the breadth and complexity of pathogens identified with this test, not revealed in previously published studies.

### Data availability.

The Karius Test is a proprietary laboratory-developed test, and thus there are several limitations on data availability that we declare here. Karius is unable to share the raw sequencing data underlying the test results evaluated here due to issues relating to patient privacy and consent. Additionally, the methodological details that can be provided to others to replicate the findings are limited due to intellectual property concerns. Taking these two limitations into account, summarized data used to reach the conclusions in this study are included in the manuscript and supplemental material.
