# Supplementary material for: Plasma Microbial Cell-Free DNA Sequencing from over 15,000 Patients Identified a Broad Spectrum of Pathogens
Source: J Clin Microbiol. 2023 Jul 13;61(8):e01855-22. doi: 10.1128/jcm.01855-22 (PMC10446866; doi:10.1128/jcm.01855-22)
Supplement: Supplemental file 1 — Tables S1, S3, and S4. Download jcm.01855-22-s0001.docx, DOCX file, 0.04 MB [file jcm.01855-22-s0001.docx]

**Supplemental Table 1.** Evolution of the Karius Test wet bench procedures and analytical pipeline over the study period, Apr 2018–Sept 2021.

| **Karius Test Version** | **Implementation date** | **Improvement** |
| --- | --- | --- |
| 3.1 | February, 2018 | Quantitative results; changed chemistry to reduce contamination and low-GC bias. |
| 3.2 | May, 2018 | Added 83 taxa to database; added new genomic sequences to pathogen database. Also, made performance improvements to reduce run time. |
| 3.4 | January, 2019 | Reduced sequencing read length from 76 to 56 bp; reducing analysis time by 2 hr. Improved accuracy of quality control filters. |
| 3.6 | October, 2019 | Extended set of “healthy” samples used for reference interval from 167 to 684. Updated taxon names after taxonomy review. |
| 3.7 | July, 2020 | Added 125 taxa to reported microbes; updated the taxon names for 128 taxa after taxonomy review. Data-driven extension of database to capture emerging infectious diseases and known existing gaps. |
| 3.8 | January,2021 | Update to list of reportable microbes. |
| 3.9 | May, 2021 | Sensitivity improved for 40 *Aspergillus* spp. |
| 3.10 | August, 2021 | Sensitivity improved for 183 high relevance taxa. Introduction of an algorithm to reduce unnecessary requeues. Shorter time to results due to fewer requeue events. |

**Supplemental Table 3.** Reports with co-detections of *Legionella* spp., *Nocardia* spp., and *Mycobacterium* spp. and their resolution, April 2018–September 2021.

| **Sample no.** | **Genus** | **Taxon name** | **MPM** | **Resolution^1^** |
| --- | --- | --- | --- | --- |
| 1 | ***Legionella* spp.** | ***L. brunensis*** | 401 | Single sp. not in database |
|  |  | ***L. hackeliae*** | 270 |  |
|  |  | Epstein-Barr virus (EBV) | 1,405 |  |
| 2 | ***Legionella* spp.** | ***L. feeleii*** | 78,508 | Single sp. not in database |
|  |  | ***L. tunisiensis*** | 76,445 |  |
|  |  | *Aspergillus fumigatus* | 594 |  |
|  |  | *Encephalitozoon hellem* | 739 |  |
| 3 | ***Nocardia* spp.** | ***N. alba*** | 222 | Single sp. not in database |
|  |  | ***N. caishijiensis*** | 334 |  |
|  |  | ***N. coubleae*** | 495 |  |
|  |  | ***N. ignorata*** | 182 |  |
|  |  | ***N. thailandica*** | 171 |  |
|  |  | Human adenovirus D | 747 |  |
|  |  | *Prevotella melaninogenica* | 283 |  |
| 4 | ***Nocardia* spp.** | ***N. exalbida*** | 17,384 | Single sp. not in database |
|  |  | ***N. gamkensis*** | 22,906 |  |
| 5 | ***Nocardia spp.*** | ***N. exalbida*** | 2,153 | Single sp. not in database |
|  |  | ***N. gamkensis*** | 1,871 |  |
| 6 | ***Nocardia* spp.** | ***N. exalbida*** | 182 | Single sp. not in database |
|  |  | ***N. gamkensis*** | 242 |  |
|  |  | *BK polyomavirus* | 81 |  |
|  |  | *Pseudomonas aeruginosa* | 498 |  |
| 7 | ***Nocardia* spp.** | ***N. elegans*** | 131 | Single sp. not in database |
|  |  | ***N. nova*** | 440 |  |
|  |  | *Actinomyces oris* | 150 |  |
|  |  | *Bacteroides vulgatus* | 215 |  |
|  |  | *Corynebacterium striatum* | 1,590 |  |
|  |  | *Enterococcus faecalis* | 296 |  |
|  |  | *Lactobacillus fermentum* | 117 |  |
|  |  | *Lomentospora prolificans* | 215 |  |
|  |  | *Prevotella buccae* | 138 |  |
|  |  | *Prevotella oralis* | 209 |  |
|  |  | *Rothia mucilaginosa* | 874 |  |
|  |  | *Staphylococcus aureus* | 364 |  |
|  |  | *Streptococcus salivarius* | 1,032 |  |
|  |  | *Veillonella parvula* | 122 |  |
| 8 | ***Nocardia* spp.** | ***N. africana*** | 1,745 | Single sp. not in database |
|  |  | ***N. elegans*** | 6,314 |  |
|  |  | ***N. nova*** | 3,945 |  |
|  |  | Cytomegalovirus (CMV) | 12,597 |  |
| 9 | ***Nocardia* spp.** | ***N. abscessus*** | 619 | Single sp. not in database |
|  |  | ***N. arthritidis*** | 317 |  |
|  |  | ***N. asiatica*** | 303 |  |
| 10 | ***Nocardia* spp.** | ***N. elegans*** | 1,556 | Single sp. not in database |
|  |  | ***N. nova*** | 2,720 |  |
| 11 | ***Nocardia* spp.** | ***N. elegans*** | 1,652 | Single sp. not in database |
|  |  | ***N. nova*** | 1,572 |  |
| 12 | ***Nocardia* spp.** | ***N. exalbida*** | 16,286 | Single sp. not in database |
|  |  | ***N. gamkensis*** | 14,660 |  |
|  |  | JC polyomavirus | 337 |  |
| 13 | ***Nocardia* spp.** | ***N. coubleae*** | 220 | Single sp. not in database |
|  |  | ***N. ignorata*** | 264 |  |
|  |  | ***N. thailandica*** | 151 |  |
|  |  | Cytomegalovirus (CMV) | 81 |  |
|  |  | Human adenovirus D | 692 |  |
| 14 | ***Nocardia* spp.** | ***N. aobensis*** | 1,122 | Single sp. not in database |
|  |  | ***N. kruczakiae*** | 3,074 |  |
|  |  | ***N. violaceofusca*** | 1,514 |  |
|  |  | *Aspergillus fumigatus* | 4,204 |  |
|  |  | BK polyomavirus | 2,260 |  |
|  |  | Epstein-Barr virus (EBV) | 49 |  |
|  |  | JC polyomavirus | 61 |  |
| 15 | ***Mycobacterium* spp.** | ***M. brisbanense*** | 20 | True detections of 3 different spp. |
|  |  | ***M. mucogenicum*** | 21 |  |
|  |  | ***M. obuense*** | 28 |  |
|  |  | *Stenotrophomonas acidaminiphila* | 36 |  |
| 16 | ***Mycobacterium* spp.** | ***M. chubuense*** | 61 | Single sp. not in database |
|  |  | ***M. elephantis*** | 124 |  |
|  |  | ***M. flavescens*** | 112 |  |
|  |  | ***M. goodii*** | 54 |  |
|  |  | ***M. holsaticum*** | 108 |  |
|  |  | ***M. phlei*** | 100 |  |
|  |  | *Neisseria meningitidis* | 62 |  |
| 17 | ***Mycobacterium* spp.** | ***M. avium complex (MAC)*** | 441 | Single sp. not in database |
|  |  | ***M. celatum*** | 135 |  |
|  |  | ***M. kyorinense*** | 194 |  |
|  |  | Epstein-Barr virus (EBV) | 155 |  |
|  |  | *Prevotella melaninogenica* | 398 |  |
|  |  | *Rothia mucilaginosa* | 913 |  |
|  |  | *Veillonella dispar* | 236 |  |
|  |  | *Veillonella parvula* | 122 |  |
| 18 | ***Mycobacterium* spp.** | ***M. avium complex* (MAC)** | 8,039 | Single sp. not in database |
|  |  | ***M. chimaera*** | 2,410 |  |
| 19 | ***Mycobacterium* spp.** | ***M. avium complex* (MAC)** | 104 | Single sp. not in database |
|  |  | ***M. chimaera*** | 104 |  |
| 20 | ***Mycobacterium* spp.** | ***M. complex* (MAC)** | 36,867 | Single sp. not in database |
|  |  | ***M. chimaera*** | 36,862 |  |

^1^Based on the pattern we expected to see in the alignments in three hypothetical scenarios: (i) a true co-detection of two or more species in the Karius database, (ii) a single species in the Karius database, and (iii) a single species not in the Karius database. In each scenario, we expected certain relative proportions of reads aligning uniquely to each species in the database: those shared among closely related species, those shared among distantly related species, and those aligning more broadly across the entire genus or family. We also considered the BLAST percent identity of the alignments for each scenario and subset of reads.

**Supplemental Table 4**. Studies reporting performance measures of mcfDNA sequencing compared with standard of care (SOC) microbiology test results in identifying clinically adjudicated causes of infection across a broad range of patient populations and microbes.

| **Study (patient population)** | **No. patients** | **Performance measures** |
| --- | --- | --- |
| Blauwkamp, et al. (12)  (Sepis) | 350 | mcfDNA PPA^1^ 92.9% and NPA^2^ 62.7% compared with composite microbiology reference standard. The diagnostic yields for plasma mcfDNA, composite microbiology tests, and blood culture were, 49%, 40%, and 18%, respectively. |
| Benamu, et al. (14) (Febrile neutropenia in cancer) | 55 | At time of fever onset (T1), PPA and NPA were 90% (9/10) and 31% (14/45), respectively; 61% of mcfDNA detections were polymicrobial. Following adjudication, mcfDNA sensitivity and specificity were 85% (41/48) and 100% (14/14), respectively. Calculated time to diagnosis was generally shorter with mcfDNA (87%). At T1, SOC was positive in 42% (20/48) patients with adjudicated infection as the etiology of FN; 18% (10/55) had positive blood culture results. |
| Wilke, et al. (58)  (Hospitalized pediatric patients) | 100 | Composite reference standard (per organism/per test adjudication) with SOC tests +/- 3 days of mcfDNA collection.  PPA 89.6% / NPA 52.3% (SOC PPA 42% / NPA 83.9%);  65.7% positive mcfDNA deemed clinically relevant. |
| Rossoff, et al. (13)  (Hospitalized pediatric patients) | 79 | All patients: sensitivity 92%/specificity 64% (SOC 77%/89%); immunocompromised patients: sensitivity 93%/specificity 59% (SOC 76%/92%); patients with invasive procedures: mcfDNA diagnostic yield 87% (SOC 67%). |
| Eichenberger, et al. (8) (Endocarditis) | 30 | Both mcfDNA and blood cultures achieved a sensitivity of 87%. The median duration of positivity from antibiotic initiation was 38.1 days for mcfDNA versus 3.7 days for blood culture. mcfDNA levels significantly declined (−0.3 MPM log10 units, 95% credible interval −0.45 to −0.14) after surgical source control was performed. |
| Degner, et al. (59)  (Hematopoietic stem cell transplant recipients) | 69 | Systematic literature review and meta-analysis of the diagnostic value of mcfDNA sequencing (6 studies). PPA of 90% and NPA of 75% compared with SOC testing. |

^1^Positive precent agreement.

^2^Negative percent agreement.
